# Cortical Evoked Potentials in Children of Diabetic Mothers

**DOI:** 10.1155/2011/640535

**Published:** 2011-10-01

**Authors:** Mario Brinciotti, Angela Napoli, Antonio Mittica, Olimpia Bitterman, Maria Matricardi

**Affiliations:** ^1^Department of Pediatrics and Child Neuropsychiatry, Faculty of Medicine and Dentistry, Sapienza University of Rome, 00185 Rome, Italy; ^2^Department of Clinical and Molecular Medicine, Faculty of Medicine and Psychology, Sapienza University of Rome, 00189 Rome, Italy

## Abstract

Type 1 diabetic mothers' infants show a delay of visual evoked potential (VEP) significantly related to some parameters of poor metabolic control during pregnancy. In the present paper we analyzed the characteristics of VEPs and somatosensory evoked potentials (SEPs) recorded in 16 three-year-old type 1 diabetic mothers' children (DMC). Compared with controls (23 nondiabetic mothers' healthy matched children), DMC showed significantly delayed mean latency of VEP (P2) and SEP (P22). In 3 cases (19%), we found pathological responses (+3 SD from the mean value of controls) of VEPs and SEPs. At the age of 3 years, the offspring of type 1 diabetic mothers showed delay of cortical evoked responses in both visual and somatosensory systems.

## 1. Introduction

Evoked potentials are commonly used in clinical practice to study brain maturation and clinical disorders [1–3]. Diabetic patients frequently show abnormal evoked potentials, usually related to neuropathy, retinopathy, and poor metabolic control [4, 5]. Subclinical CNS dysfunctions have been reliably detected by evoked potentials in adult patients with uncomplicated diabetes and normal brain CT scan [6]. Moreover, evoked potentials are sensitive to drug administration effects and prenatal substance exposure [7, 8]. In previous studies [9, 10] on diabetic mothers' infants, we found a delayed mean latency of the fourth (P2) component of VEPs compared to matched healthy infants. These results were significantly related to some parameters of poor metabolic control during pregnancy in type 1 diabetic mothers' infants; on the contrary, in infants born from mothers with gestational diabetes VEPs did not show any significant relation with metabolic parameters during pregnancy, but latencies correlated with Apgar scores of perinatal distress. These features suggest that in the offspring of type 1 diabetic mothers VEP changes may be related to adverse effects due to exposure of the fetus to metabolic imbalance during intrauterine life.

These observations raise two main points: (i) whether the VEP abnormalities recorded at the age of 2 months are transient and (ii) whether the abnormalities of evoked responses are restricted to the visual system or extended to other cortical structures. To investigate both these aspects, in the present study we analyzed the characteristics of VEPs and somatosensory evoked potentials (SEPs) recorded in 3-year-old type 1 diabetic mothers' children (DMC).

## 2. Materials and Methods

### 2.1. Sample

We studied VEPs and SEPs of 16 three-year-old children (11 females, 5 males, mean age 2.6 ± 0.9 years), whose mothers suffered of type 1 diabetes. VEPs had already been recorded at the age of two months (24 infants, 8 missing cases; 33% attrition rate between 2 months and 3 years). VEPs and SEPs were obtained according to the American Electroencephalographic Society guidelines [11] with electrodes placement based on the International 10–20 System. Recordings were performed without knowledge of mothers' antepartum metabolic status and infants' perinatal history. Informed consent was obtained from each child's parents after explaining to them the procedures' nature and purpose. A matched sample of 23 nondiabetic mothers' healthy children was used as control.

### 2.2. VEP Recording

VEPs were recorded in a partially darkened room (mean background light 0.15 ft-Lamberts; dark adaptation for 20 minutes) in awake condition (without sedation). The state of alertness was carefully checked during the entire recording session. VEPs were elicited by binocular stimulation with a stroboscopic unpatterned flash (white light; intensity 0.3 Joule; frequency 1 Hz) placed about 25 cm from the eyes. Responses were recorded from silver-silver chloride electrodes applied to the occipital region, using a four-channel montage (O1-Fz, Oz-Fz, O2-Fz, Fz-M1; M2 as ground). At least two trials of 100 artifact-free responses (automatic artifact rejection; amplitude threshold <20% of rejected traces) were recorded within 512 ms after stimulus.

### 2.3. SEP Recording

SEPs were obtained in conscious subjects (without sedation) checking the alertness during the entire recording session. Responses were elicited by electrical stimulation applied on the left median nerve at the wrist using a constant current square wave pulse (0.1 ms width, cathode proximal) at a repetition rate of 4 Hz. The stimulus intensity was regulated to produce a small thumb twitch. Cortical responses were recorded by surface silver-silver chloride electrodes placed on the contralateral parietal and frontal areas with two channel montage with an active electrode on C4′ (2 cm posterior to C4 in the International 10–20 System) referenced to Fz, and an active electrode on F4 referenced to C3′. At least two trials of 250 artifact-free samples (automatic artifact rejection; amplitude threshold <20% of rejected traces) were recorded with an analysis time of 100 ms.

All reproducible peaks of VEPs and SEPs were identified and labelled according to the American Electroencephalographic Society guidelines [11]. Peak latencies and peak-to-peak amplitudes of all components were measured but only the most stable components were used for statistical comparisons (III, IV, and V for VEPs; N20 and P22 for SEPs). Pathological responses were considered when latency value was more than 3 SD from the mean value of controls.

### 2.4. Statistical Analysis

Means and limits were calculated adopting tolerance limit of 99% with a confidence of 95%. To obtain normative data for VEPs and SEPs, the distribution of observed values was previously examined by the Shapiro-Will's goodness of fit for skewness and/or kurtosis. Statistical analysis was performed by software (STATISTICA 9.1-StatSoft, Inc., OK, USA), using ANOVA with post hoc comparison (Bonferroni test for multiple comparisons), nonparametric tests, and *χ* ^2^ with Yates correction when appropriate. Statistical significance was defined as *P* < 0.05.

## 3. Results

DMC showed mean latency of all VEP components significantly increased compared with controls at the age of two months and the forth (P2) component was still delayed at the age of three years (Table 1). No differences were found for VEP amplitudes.

The P22 component of SEP was significantly delayed compared with controls (Table 2) whereas no differences were found for N20 latency and for amplitudes of both waves. Pathological responses of VEPs and SEPs were found in 3 cases (19%).

## 4. Discussion

At the age of two months, type 1 diabetic mothers' offspring showed that all VEP latencies were increased than controls, and this finding is still evident at the age of three years for the fourth VEP component (P2). The cortical origin of this wave is well documented by multichannel scalp recordings and clinical studies [12, 13]. According to the hypothesis of subcortical-cortical development, during the first postnatal weeks, the visual system is under the prevailing control of subcortical centres, afterwards the primary geniculocalcarine cortical system assumes the major control, due to synaptogenesis and myelination [14]. Visual processes continue to mature during childhood and these changes may be well documented by VEP recording, as a decrease in latency, an increase in amplitude, and a development of the waveform [15, 16]. Data of VEPs in our sample suggest the occurrence of adverse effects of maternal diabetes on visual system especially at cortical level; these effects are not limited to the neonatal period but appear to be persistent, at least in the first 3 years of age.

Regarding SEPs, we found a delay in the P22 but not in the N20 component. These waves represent the initial response of the primary somatosensory cortex to stimulation of the upper extremity. Clinical and experimental data suggest differential generators for N20 and P22 (N20 generator in the postcentral gyrus-S1, Brodmann area 3b; P22 generator assigned either to the area 4 in the precentral gyrus either to area 1 in the postcentral gyrus) [17, 18]. Our data are consistent with these observations, and they support the possibility of separate functional testing of Brodmann areas, as recently reported [19]. Moreover, the delay of the P22 component in the offspring of diabetic mothers suggests that effects of metabolic imbalance during the intrauterine life are not confined to the visual system but act more diffusely.

All these data confirm previous studies on the adverse effects of maternal diabetes on offspring's brain development [20–23]. Cortical dysfunctions could be related to poor metabolic control, as suggested by clinical and experimental studies, with subtle negative effects on the offspring's CNS [24–26].

Finally, in the present study we found pathological responses of both VEPs and SEPs in 3 children, currently asymptomatic. Central nervous system degeneration is a well-known long-term complication in diabetic patients, and it is possible to reveal this involvement in an early asymptomatic stage by using evoked potentials [27, 28]. Neuropsychological deficits have been noted in children with type 1 diabetes [29], suggesting the occurrence of subtle negative effects related to diabetes on cognitive development in school-age children. Recurrent episodes of hypoglycaemia as well extended periods of hyperglycemia have been reported as possible causes of brain dysfunction in poorly controlled diabetic patients [30, 31]. Moreover, the recent concept of developmental programming postulates long-term detrimental effects on adult health due to nutritional imprinting during critical developmental periods; according to this finding, alterations and/or modifications in nutrients supply during fetal and neonatal life may be associated with abnormal growth patterns, resulting in the development of future diseases [32–36]. To clarify the clinical significance of the abnormal evoked responses found in our sample, we are carrying out a clinical and neurophysiologic followup of these children.

## 5. Conclusions

Type 1 diabetes mothers' offspring showed a significant delay of cortical evoked responses to both visual and somatosensory stimulation compared with controls. These results do not seem to be transient, since the delay of VEP found in the neonatal period is still present at the age of three years. The recording of evoked potentials may be proposed as a useful tool of investigation since it is particularly sensitive to highlight functional abnormalities of the CNS.

## Figures and Tables

**Table 1:** Comparison between DMC and controls for VEP latencies.

Age	VEP components	DMC			Controls			P*
		Latency (ms)			Latency (ms)			
		Mean ± SD	Confidence interval		Mean ± SD	Confidence interval		
			−99%	+99%		−99%	+99%	
2 months	Right (O2)							
	III	136.2 ± 29.9	114.2	158.2	109.0 ± 31.1	89.8	129.3	<0.01
	IV	190.9 ± 33.4	166.3	215.5	156.6 ± 30.8	137.4	175.7	<0.01
	V	275.8 ± 50.1	238.9	312.8	223.6 ± 47.1	194.4	252.9	<0.01
	Left (O1)							
	III	132.3 ± 29.6	110.5	154.1	108.4 ± 31.6	88.7	128.0	<0.05
	IV	187.8 ± 37.1	160.4	215.1	156.7 ± 31.7	137.0	176.4	<0.01
	V	267.2 ± 47.3	232.3	302.1	220.1 ± 50.0	189.0	251.1	<0.01

3 years	Right (O2)							
	III	71.53 ± 15.2	62.3	78.4	67.63 ± 9.0	61.5	74.0	NS
	IV	105.53 ± 13.1	98.7	114.5	99.96 ± 8.8	93.8	106.1	<0.05
	V	149.03 ± 36.2	133.2	174.2	149.05 ± 22.5	133.5	165.5	NS
	Left (O1)							
	III	74.47 ± 13.4	65.8	81.3	68.02 ± 9.7	62.0	74.0	NS
	IV	106.13 ± 14.0	98.4	116.0	98.69 ± 10.2	91.8	105.6	<0.05
	V	151.12 ± 43.3	135.9	180.1	150.0 ± 22.4	132.8	167.3	NS

*ANOVA (post hoc comparison with correction for multiple comparisons—Bonferroni test).

**Table 2:** Comparison between DMC and controls for SEP latencies.

SEP components	DMC			Controls			P*
	Latency (ms)			Latency (ms)			
	Mean ± SD	Confidence interval		Mean ± SD	Confidence interval		
		−99%	+99%		−99%	+99%	
N20	18.11 ± 1.98	14.20	22.02	17.55 ± 1.42	13.87	21.23	NS
P22	24.86 ± 2.38	15.22	34.50	21.71 ± 1.57	14.12	29.30	<0.001

*ANOVA (post hoc comparison with correction for multiple comparisons—Bonferroni test).
